# Total Psoas Area Predicts Complications following Radical Cystectomy

**DOI:** 10.1155/2015/901851

**Published:** 2015-12-21

**Authors:** Timothy D. Lyon, Nicholas J. Farber, Leo C. Chen, Thomas W. Fuller, Benjamin J. Davies, Jeffrey R. Gingrich, Ronald L. Hrebinko, Jodi K. Maranchie, Jennifer M. Taylor, Tatum V. Tarin

**Affiliations:** ^1^Department of Urology, University of Pittsburgh, Pittsburgh, PA, USA; ^2^Division of Urology, Rutgers Robert Wood Johnson Medical School, New Brunswick, NJ, USA; ^3^University of Pittsburgh School of Medicine, Pittsburgh, PA, USA; ^4^Department of Urology, Baylor College of Medicine, Houston, TX, USA

## Abstract

*Purpose*. To determine whether total psoas area (TPA), a simple estimate of muscle mass, is associated with complications after radical cystectomy.* Materials and Methods*. Patients who underwent radical cystectomy at our institution from 2011 to 2012 were retrospectively identified. Total psoas area was measured on preoperative CT scans and normalized for patient height. Multivariable logistic regression was used to determine whether TPA was a predictor of 90-day postoperative complications. Overall survival was compared between TPA quartiles.* Results*. 135 patients were identified for analysis. Median follow-up was 24 months (IQR: 6–37 months). Overall 90-day complication rate was 56% (75/135). TPA was significantly lower for patients who experienced any complication (7.8 cm^2^/m^2^ versus 8.8 cm^2^/m^2^, *P* = 0.023) and an infectious complication (7.0 cm^2^/m^2^ versus 8.7 cm^2^/m^2^, *P* = 0.032) than those who did not. On multivariable analysis, TPA (adjusted OR 0.70 (95% CI 0.56–0.89), *P* = 0.003) and Charlson comorbidity index (adjusted OR 1.34 (95% CI 1.01–1.79), *P* = 0.045) were independently associated with 90-day complications. TPA was not a predictor of overall survival.* Conclusions*. Low TPA is associated with infectious complications and is an independent predictor of experiencing a postoperative complication following radical cystectomy.

## 1. Introduction

Bladder cancer is a common genitourinary malignancy, with an estimated 72,570 new cases reported in the United States in 2013 [1]. Radical cystectomy (RC) with pelvic lymph node dissection remains the standard of care for muscle-invasive bladder cancer. RC is a morbid procedure, with readmission rates approaching 27% and 90-day complication rates as high as 64% [2, 3]. A number of preoperative risk factors portend risk of morbidity and mortality following RC, including age, comorbidities, functional status, and nutritional deficiency [4–6].

Multiple somatometric and serologic markers have been used to quantify nutritional deficiency including body mass index (BMI), weight loss, serum albumin, and skeletal muscle mass [5–7]. Perhaps the least well studied of these is sarcopenia, or loss of total body skeletal muscle mass. Low skeletal muscle index (SMI) is associated with decreased cancer-specific and overall survival following RC [8] but obtaining this measurement requires specialized imaging software and cannot be efficiently determined in a clinical setting. Recent work has investigated whether total psoas area (TPA), which can be more easily determined, provides a reliable estimate of muscle mass and carries the same prognostic value as SMI [9–11]. Smith et al. found an association between low TPA and increased major complication rate following RC in women [12], but this finding requires further validation.

To clarify whether TPA is a useful prognostic marker following RC, we measured TPA on preoperative computed tomography (CT) scans for patients who underwent RC at our institution over a two-year period and compared these to patient outcomes.

## 2. Materials and Methods

### 2.1. Patients and Study Design

Following institutional review board approval, we performed a retrospective chart review to identify all patients who underwent RC for bladder cancer at the University of Pittsburgh Medical Center from Jan. 1, 2011, through Dec. 31, 2012. Patients were excluded if a CT scan performed within 30 days prior to RC was not available. RC was performed by one of eight high-volume surgeons in our department. All pathology specimens were reviewed by a dedicated genitourinary pathologist.

Demographic, pathologic, and outcome data were collected for each patient. Complications were categorized according to the Clavien-Dindo classification [13]. The primary outcome was 90-day overall complication rate. Secondary outcomes included major complications (Clavien grades III–V), infectious complications, wound complications, 90-day all-cause mortality, and overall survival. Classification as an infectious complication required a positive culture and treatment with antibiotics, and wound complications referred to fascial dehiscence, enterocutaneous fistula, or incarcerated incisional hernia. Follow-up was determined as of 4/19/2015, with patients censored at the date of their last confirmed visit to our institution or documented death.

### 2.2. TPA Measurement

TPA was measured at the L3 vertebral level on CT scan by a single investigator blinded to patient outcomes. Measurements were taken on the first image where both transverse processes were visible while traveling in the craniocaudal direction. Images were viewed using iSite PACS radiology software (Phillips, Amsterdam, Netherlands). Cross-sectional area of each psoas muscle was summed. To normalize TPA for body size, each TPA was then divided by the individual's body surface area (BSA), calculated from the height and weight recorded as a routine part of each patient's hospital admission, using the formula BSA = √[(height (cm) × weight (kg))/3600].

### 2.3. Statistical Analysis

Patients were grouped according to whether or not they experienced a 90-day complication for purposes of comparison. Means with standard deviations (SD) are reported for parametric data and medians with interquartile ranges (IQR) are reported for nonnormal data. TPA was evaluated as a continuous variable. Categorical variables were compared using *χ* ^2^ and Fisher's exact tests as appropriate. Means were compared using Student's *t*-test and medians using the Mann-Whitney *U* test. A multivariable logistic regression model was used to identify predictors of experiencing a postoperative complication while controlling for patient and disease characteristics known to be associated with adverse outcomes after RC. The Kaplan-Meier method was used to examine overall survival stratified by TPA quartile and compared using the log rank test. Data was analyzed using SPSS software, version 20 (IBM Corp., Armonk, NY). Statistical significance was defined at the *P* < 0.05 level using two-tailed tests.

## 3. Results

We identified 135 patients for analysis. Mean age was 69 years and 15% of patients (20/135) received neoadjuvant chemotherapy. Other than TPA, there were no significant differences between groups for any measured clinical or pathologic variables (Table 1).

Seventy-five patients experienced 102 postoperative complications (Table 2). Overall 90-day complication rate was 56% (75/135). Forty patients (30%) experienced a Clavien grade I-II complication, and major complications (Clavien grades III–V) occurred in 35 patients (26%). Infectious complications occurred in 22/135 patients (16%) and wound complications in 13/135 (9.4%). On bivariate analysis, infectious complications were not significantly associated with patient age (*P* = 0.39), smoking status (*P* = 0.35), Charlson comorbidity index (*P* = 0.45), EBL (*P* = 0.88), operative time (*P* = 0.06), or diversion type (*P* = 0.14); however, median BMI was higher in patients who had an infectious complication (28.7 kg/m^2^ versus 25 kg/m^2^, *P* = 0.01) and a wound complication (29.5 kg/m^2^ versus 25.7 kg/m^2^, *P* = 0.046). Wound complications were not significantly associated with age (*P* = 0.6), smoking status (*P* = 0.37), Charlson comorbidity index (*P* = 0.42), EBL (*P* = 0.39), operative time (*P* = 0.09), or diversion type (*P* = 0.06). Ninety-day mortality was 7.4% (10/135).

Median TPA was significantly lower in patients that experienced a 90-day complication (7.8 cm^2^/m^2^ versus 8.8 cm^2^/m^2^, *P* = 0.023) and an infectious complication (7.0 cm^2^/m^2^ versus 8.7 cm^2^/m^2^, *P* = 0.032) than in patients who did not. There was no significant difference in TPA between groups for major complications (8.1 cm^2^/m^2^ versus 8.5 cm^2^/m^2^, *P* = 0.64), wound complications (7.3 cm^2^/m^2^ versus 8.6 cm^2^/m^2^, *P* = 0.43), or 90-day mortality (7.6 cm^2^/m^2^ versus 8.5 cm^2^/m^2^, *P* = 0.34).

Multivariable analysis investigating factors associated with experiencing a 90-day complication was performed (Table 3). After adjustment for potential cofounders, independent predictors of postoperative complications included TPA (adjusted OR 0.70 (95% CI 0.56–0.89), *P* = 0.003) and Charlson comorbidity index (adjusted OR 1.34 (95% CI 1.01–1.79), *P* = 0.045).

**Figure 1:**
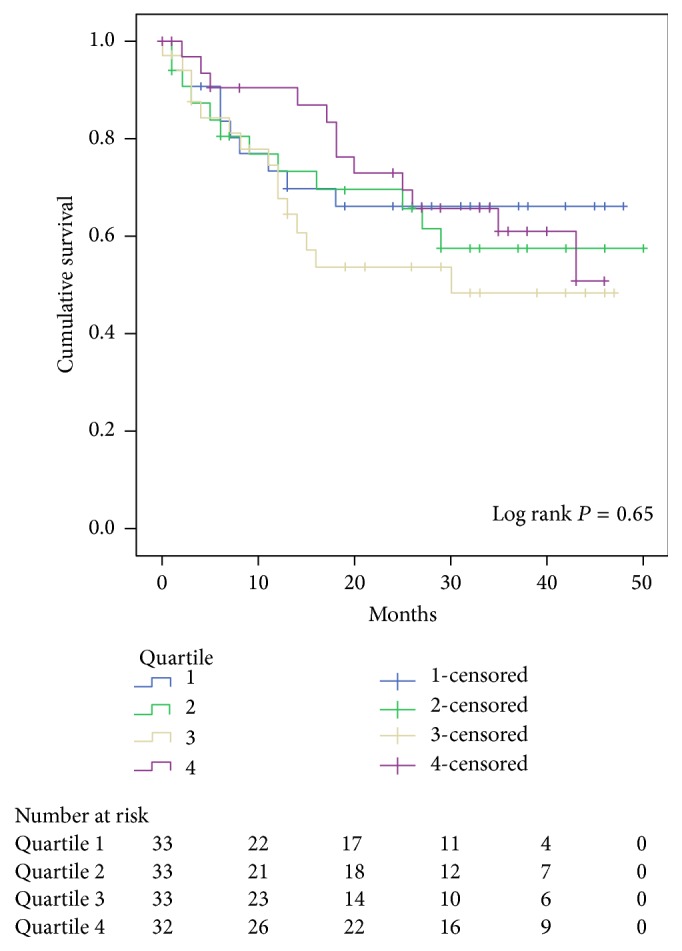
Overall survival stratified by TPA quartile.

At a median follow-up of 24 months (IQR: 6–37 months), overall survival was 62% (86/135). When analyzed by quartile, TPA was not a significant predictor of overall survival (*P* = 0.65). Median survival was 34.3 months (95% CI 27.3–41.3), 33.9 months (26.7–41.2), 28.4 months (95% CI 21.6–35.2), and 34.6 months (95% CI 29.2–40.1) for TPA quartiles 1–4, respectively (Figure 1).

## 4. Discussion

Identifying bladder cancer patients at high risk of postsurgical complications is an important target for improving outcomes after cystectomy. In this study we sought to determine whether TPA, an estimate of skeletal muscle mass, was associated with 90-day outcomes following RC. On multivariable analysis, our data revealed that TPA (adjusted OR 0.70, *P* = 0.003) and Charlson comorbidity index (adjusted OR 1.34, *P* = 0.045) were independent predictors of a postsurgical complication. The odds ratio for TPA corresponds to a 30% decrease in complication risk for each 1 cm^2^/m^2^ increase in TPA. TPA was also associated with infectious complications (*P* = 0.032) on bivariate analysis, as was BMI (*P* = 0.01). Unfortunately, due to low event rate a multivariable risk adjustment for infectious complications could not be performed. However, these results are important as they suggest that TPA may be able to identify patients most at risk of complications following RC, potentially improving preoperative risk stratification.

Traditionally associated with aging, sarcopenia, or low skeletal muscle mass, is a prognostic marker of poor overall nutritional status and has been associated with increased mortality in elderly hospitalized patients [7, 14–16] as well as with adverse outcomes in patients undergoing surgery for gastrointestinal malignancies [17–21]. The most well-validated measure of muscle mass is the skeletal muscle index (SMI), obtained by measuring the cross-sectional area of all skeletal muscles including the rectus abdominis; internal, external, and lateral obliques; psoas; quadratus lumborum; and erector spinae muscles at the L3 vertebral level and normalizing for height in m^2^ [22]. Multiple studies have validated the predictive value of skeletal muscle area at the L3 or L4 vertebrae on CT scan as a surrogate measure of whole-body skeletal muscle mass in both healthy and cancer populations [23, 24]. In a cohort of 205 patients who underwent RC, Psutka et al. used validated gender-specific SMI cutoffs to define sarcopenia for each gender and found that SMI was an independent predictor of cancer-specific and overall survival at a median follow-up of 6.7 years [8]. In contrast to these results, we found no difference in overall survival in our patients when compared by TPA quartile (*P* = 0.65); however, our median follow-up was only 2 years, and it is possible that with longer follow-up a survival difference would be uncovered.

Total psoas area has also been linked to complications following radical cystectomy; Smith et al. reviewed a series of 200 patients and found that low TPA was a predictor of 30-day major complications in women, but not in men [12]. Similarly, we demonstrated an association between TPA and overall complications (*P* = 0.003) and infectious complications (*P* = 0.032) after RC but did not replicate their finding of an increased risk of major complications. Two protocol differences may account for the differences in our findings. First, we evaluated 90-day instead of 30-day outcomes. Secondly, Smith et al. normalized TPA using height in m^2^, while we opted to normalize it with body surface area to better account for obesity and patient size than height alone [12, 25]. Replication of our findings will be required to determine which of these approaches is optimal.

One potential advantage of using TPA over SMI for the quantification of sarcopenia in surgical patients is the ease with which it can be obtained. Psoas area can be easily and efficiently measured on CT images, a modality very familiar to most surgeons, in an outpatient setting. Further, TPA has been proposed as the surrogate marker best suitable for studying sarcopenia in diseased populations, as the psoas muscle reflects changes of nutritional deficiency in chronically ill patients but not acutely ill ones [14, 26]. Our TPA measurement technique differed slightly from those previously described in that we did not use HU criteria to automatically subtract fatty infiltration from muscle area. Our rationale for doing so was to determine whether a rapidly obtainable clinical measurement could be used in preoperative risk stratification and we did not want to rely on imaging software not available to the average urologist. In centers where SMI is not routinely reported or where radiologists are unfamiliar with the technique, urologists could more easily calculate TPA than SMI themselves, leading to greater clinical utility.

Nutritional deficiency has been linked to inferior outcomes after RC but has been variably defined, using preoperative albumin levels, body mass index, weight loss, and sarcopenia [4–6, 8, 12]. Confusion exists as to how best to use these markers. Establishing an optimal method of quantifying malnutrition in bladder cancer patients is important, as it may be a factor upon which clinicians can intervene to improve surgical quality. Malnutrition is known to alter the immune response to surgical stress and increase susceptibility to postoperative infections [27, 28]. Specifically, sarcopenia has been shown to predict a greater risk of experiencing serious infections after liver transplantation [9] and of acquiring a nosocomial infection [29]. TPA may be a useful marker in bladder cancer patients as well based on our observed difference in postoperative infection rate. Postcystectomy infectious complications are known to increase perioperative mortality, length of stay, and hospital costs [30]. Preoperative supplementation with immunonutrient high-arginine shakes has been shown to reduce infectious complications and length of stay following gastrointestinal surgery [31–33]. Our results imply that TPA may be able to identify patients at the highest risk of post-RC infectious complications that could potentially benefit from preoperative immunonutrient supplementation or other such targeted interventions, and we are currently enrolling patients at our center in a prospective trial examining whether such supplementation can influence outcomes after RC. Until trial results are available, however, we cannot yet definitively state that preoperative immunonutrient shake supplementation can improve post-RC outcomes in malnourished patients.

Several limitations exist in this study. First, our analysis is retrospective in nature and subject to problems inherent to this approach. The sample size is relatively small, which limits statistical power and the generalizability of our findings. Median follow-up was 2 years, and therefore we were unable to determine whether TPA can predict long-term oncologic outcomes. Subjects are predominantly Caucasian and thus results may not be applicable to persons of other racial backgrounds. Preoperative serum albumin levels were only available for 54% of our cohort, and due to missing data we did not include albumin in our multivariable model despite the fact that low albumin has been previously linked to worse outcomes after cystectomy [5, 6]. For the data that was available, median serum albumin was not significantly different between patients who did and did not experience a postoperative complication, though (Table 1). Future investigations are needed to assess the relationship between TPA, albumin, and other nutritional markers to determine which can best predict patient outcomes. Further work with larger sample sizes and longer follow-up is needed to validate our findings and to better characterize gender-specific normal and abnormal TPA values in a cystectomy population.

Despite these limitations, our data suggest that patients with low TPA have a greater risk of overall and infectious complications after RC, building upon the findings of Smith and colleagues [12] and suggesting that TPA may be able to play a future role in selecting patients at the highest risk of post-RC complications. However, TPA was not associated with long-term survival in our series as has been shown with SMI [8]. On the basis of our findings it would be inappropriate to suggest that TPA be used independently for prognostication prior to RC at this time. Future work is needed to identify whether TPA could be used to predict survival and whether nutritional supplementation in patients with low TPA improves outcomes after RC. For the time being urologists interested in sarcopenia should ask radiologists to report SMI. However, future work may still prove TPA useful to guide targeted interventions to improve the quality of care for patients with bladder cancer.

## 5. Conclusion

Low total psoas area is associated with overall and infectious complications following radical cystectomy, but not overall survival. TPA may prove useful as a biomarker to identify patients at the highest risk of post-RC complications.

## Figures and Tables

**Table 1:** Patient characteristics.

	90-day complication	No complication	*P* value
	*n* = 75	*n* = 60	
Age, years, mean ± SD	68.5 ± 9.9	69.1 ± 9.4	0.71
Gender (%)			0.99
M	60 (80)	48 (80)	
F	15 (20)	12 (20)	
Race (%)			0.37
White	69 (92)	58 (97)	
Nonwhite	6 (8.0)	2 (3.3)	
Smoker (%)	51 (68)	37 (62)	0.56
BMI, kg/m^2^, median (IQR)	26.2 (22.5–30.4)	25.3 (22.8–29.0)	0.45
Serum albumin^*∗*^, g/dL, median (IQR)	3.7 (3.0–4.0)	3.8 (3.4–4.0)	0.22
Charlson comorbidity index			0.11
0-1	28 (37)	30 (50)	
2-3	26 (35)	24 (40)	
>3	21 (28)	6 (10)	
Neoadjuvant chemotherapy (%)	13 (17)	7 (12)	0.36
L3 TPA, cm^2^/m^2^, median (IQR)	7.8 (6.6–9.5)	8.8 (7.5–10.1)	0.023
Diversion type (%)			0.69
Neobladder	21 (28)	18 (30)	
Continent cutaneous	4 (5.3)	1 (1.7)	
Ileal conduit	48 (64)	41 (68)	
No diversion (anephric)	1 (1.3)	0	
Pathologic T Stage (%)			0.23
T0	1 (1.3)	2 (3.3)	
Ta	0	4 (6.7)	
Tis	8 (11)	6 (10)	
T1	5 (6.7)	8 (13)	
T2	14 (19)	9 (15)	
T3	30 (40)	22 (37)	
T4	17 (23)	9 (15)	
Node positive (%)	23 (31)	14 (23)	0.16
Operative time, min, mean ± SD	356 ± 103	334 ± 93	0.20
Estimated blood loss, mL, median (IQR)	850 (550–1075)	850 (675–1525)	0.56

SD: standard deviation, IQR: interquartile range, GFR: glomerular filtration rate, TPA: total psoas area, and mL: milliliter.

^*∗*^ *n* = 73.

**Table 2:** 90-day postoperative complications.

Complication	*n* (% of patients)
UTI/pyelonephritis	12 (8.9)
Ileus	9 (6.7)
Wound dehiscence	8 (5.9)
DVT/PE	7 (5.2)
Atrial fibrillation	6 (4.4)
Enterocutaneous fistula	5 (3.7)
Renal failure	5 (3.7)
*Clostridiumdifficile* infection	4 (3.0)
Stroke	4 (3.0)
Pelvic abscess	4 (3.0)
Small bowel obstruction	4 (3.0)
Ureteral obstruction requiring nephrostomy	3 (2.2)
Respiratory failure	3 (2.2)
PEA arrest	2 (1.5)
Ventricular tachycardia	2 (1.5)
Dysphagia	2 (1.5)
Wound infection	2 (1.5)
Hypoxia	2 (1.5)
Hemorrhage requiring transfusion	2 (1.5)
Delirium	2 (1.5)
Intestinal anastomotic leak	1 (0.7)
Pleural effusion	1 (0.7)
AV fistula dissection	1 (0.7)
Critical limb ischemia	1 (0.7)
Malignant hypertension	1 (0.7)
Splenic infarct	1 (0.7)
Ureteroenteric anastomotic stricture	1 (0.7)
Incarcerated incisional hernia	1 (0.7)
Chest pain	1 (0.7)
GI bleed	1 (0.7)
Infected pelvic hematoma	1 (0.7)
Compartment syndrome/rhabdomyolysis	1 (0.7)
Pancreatitis	1 (0.7)
Aspiration pneumonia	1 (0.7)

UTI: urinary tract infection, DVT: deep venous thrombosis, PE: pulmonary embolism, PEA: pulseless electrical activity, AV: arteriovenous, and GI: gastrointestinal.

**Table 3:** Multivariable predictors of 90-day complications.

	Univariate OR (95% CI)	*P* value	Adjusted OR (95% CI)	*P* value
Age	0.99 (0.96–1.03)	0.71	0.97 (0.92–1.02)	0.20
Gender (referent female)	1.00 (0.43–2.34)	0.99	2.27 (0.77–6.76)	0.14
Charlson comorbidity index	1.38 (1.07–1.78)	0.014	1.34 (1.01–1.79)	0.045
Neoadjuvant chemotherapy	1.59 (0.59–4.27)	0.36	1.29 (0.40–4.16)	0.67
Total psoas area	0.82 (0.69–0.97)	0.019	0.70 (0.56–0.89)	0.003
Pathologic T Stage				
T0	Referent	0.81	Referent	0.95
Ta	0.0	0.99	0.0	0.99
Tis	2.67 (0.19–36.76)	0.46	3.14 (0.12–83.74)	0.50
T1	1.25 (0.09–17.65)	0.87	1.84 (0.07–46.58)	0.71
T2	3.11 (0.25–39.54)	0.138	3.15 (0.14–73.16)	0.48
T3	2.73 (0.23–32.01)	0.43	3.92 (0.18–84.29)	0.38
T4	3.56 (0.28–44.88)	0.33	2.93 (0.13–64.55)	0.50
Node positive	1.76 (0.80–3.86)	0.16	1.68 (0.65–4.33)	0.29

OR: odds ratio, CI: confidence interval, and TPA: total psoas area.
